# Dynamics of the Fermentation Process and Chemical Profiling of Pomegranate (*Punica granatum* L.) Wines Obtained by Different Cultivar×Yeast Combinations

**DOI:** 10.3390/foods10081913

**Published:** 2021-08-18

**Authors:** Massimiliano Cardinale, Roberto Trinchera, Giuseppe Natrella, Graziana Difonzo, Carlo De Benedittis, Ilario D’amato, Marco Mascellani, Vito Michele Paradiso, Laura Rustioni

**Affiliations:** 1 Department of Biological and Environmental Sciences and Technologies, University of Salento, 73100 Lecce, Italy; massimiliano.cardinale@unisalento.it (M.C.); robertojosue.trinchera@studenti.unisalento.it (R.T.); laura.rustioni@unisalento.it (L.R.); 2 Department of Soil, Plant and Food Science, University of Bari Aldo Moro, 70126 Bari, Italy; giuseppe.natrella@uniba.it (G.N.); graziana.difonzo@uniba.it (G.D.); 3 ICS S.r.l., 73010 Veglie, Italy; debened@libero.it; 4 Togo Bay di D’amato Luigi, 73010 Porto Cesareo, Italy; Damato.ilario@gmail.com; 5 Marco Mascellani Consulenze Enologiche e Agronomiche, 73100 Lecce, Italy; info@marcomascellani.it

**Keywords:** pomegranate wine, fruit wine, *Saccharomyces* yeasts, volatile compounds, phenolic compounds, organic acids profile

## Abstract

Pomegranate (*Punica granatum* L.) is one of the historical tree crops in the Mediterranean region and is nowadays commercialized for its beneficial properties in the form of fruits, juice, jams and, in some East countries, as fermented juice (pomegranate wine). However, pomegranate wines are not established as a common beverage in Western countries. In this work, we produced pomegranate wines using two cultivars and two yeasts (*Saccharomyces cerevisiae* strain Clos and *S. cerevisiae* ex-*bayanus* strain EC1118) with contrasting characteristics. A comprehensive chemical profile of the wines was obtained. Notable differences were observed in the function of the cultivars and the yeasts. Different cultivar×yeast combinations provided wines with clearly different chemical profiles and specific features in the patterns of organic acids, phenolics, and volatile compounds. This highlights the opportunity to obtain tailored pomegranate wines with desired chemical profiles and, consequently, sensory properties, through management optimization of pomegranate winemaking. In this view, pomegranate wines have the potential to become an established beverage in Western countries.

## 1. Introduction

Pomegranate (*Punica granatum* L.) is widely present in historical symbolisms of the Mediterranean and Near East regions and it is cultivated worldwide for its productive and ornamental qualities [1]. Native from Iran, pomegranate was naturalized in the Mediterranean region thanks to the Phoenicians around 2000 BCE [1]. Pomegranate was found in Italy from the Archaic Period (sixth/fifth century B.C.) [2]. It was used by the Messapian people as food offerings in the Demeter and Persephone sanctuaries [3,4]. Then, Romans spread this fruit throughout their Empire [1]: they considered pomegranate as a luxury food, conserving and transporting it also outside its cultivation area [2,5].

In the symbolisms of the archaic cultures, such as Ancient Egyptian and Jewish, grapes and pomegranates were plants often cultivated in marvelous orchards to produce fruits, juices, and fermented wines [1,6,7].

Although fermentation is often used as a process to preserve juices, it seems that Romans used it to maintain pomegranates as entire fruits. Jacomet et al. [2] describes their conservation techniques, such as the storage in hermetically sealed vessels, the installation of special fruit-rooms called *pomeriums*, the external fruit treatment with plaster, wax, or clay to prevent dehydration, and the short treatment in hot salty water to make the pericarp harder. Thus, since that time, it has been well known that the fruit processing could add value to products, extending the shelf life and allowing the movement of the foods over long distances.

During the centuries, the technology evolved, and nowadays different possibilities are available for pomegranate processing. Some examples, as reported by Dhinesh and Ramasamy [8], are minimally processed pomegranate seeds, jams, marmalades, single-strength juices, jellies, juice concentrates, frozen seeds, refrigerated seeds, seeds in syrup, candied arils, arils in brandy and in vinegar, carbonated beverages, pomegranate syrup, and, last but not least, pomegranate wine.

Southern Italy, in the middle of the Mediterranean Sea, has favorable environmental conditions to grow pomegranate, and the current scenario of climate change requires the intelligent selection of tolerant crops for future agriculture. Pomegranate withstands different soils and climates, being a resilient crop adapted to arid and semi-arid regions. Furthermore, its intrinsic tolerance to biotic and abiotic stresses requires low agricultural inputs and, thus, low management costs [8]. On the other hand, Italy has a long tradition in winemaking technology and thus, both the experience and the equipment required to obtain good pomegranate fermentation products are readily available in this Country.

Nowadays, wine consumers are willing to try new sensorial experiences: this is demonstrated by the current tendency towards wines with more complex organoleptic properties. On one hand, this has led to the establishment of more diversified fermentation processes: examples of this are the use of sequential or simultaneous co-inoculation with non-*Saccharomyces* and *Saccharomyces* yeasts, in order to enrich the wine bouquet of flavors [9], and the production of spontaneously fermented wines with unique but non-reproducible characteristics (even if this might affect the microbiological stability of the product) [10]. On the other hand, new fermented beverages have been created from fruits other than grapes, and traditional fermented beverages were re-discovered [11]. 

Pomegranate wines are produced and largely consumed in a few countries, such as Armenia and Israel, as part of their food tradition. In Italy, and specifically in Sicily, there are records of “pomegranate wine” beverages called “*Sciaddé*”, a tradition that has been lost. However, it is very difficult to find scientific literature on this topic. Instead, a consistent literature exists on lactic acid bacteria fermentation of pomegranate juice [12,13], clearly indicating its potential as a fermented beverage. 

In this work, we studied the process of pomegranate juice alcoholic fermentation including the characterization of yeast growth dynamics, which, to the best of our knowledge, has been done rarely so far. Finally, we analyzed the fermented products in order to characterize them as broadly as possible, for future entry in the Italian as well as other Western countries’ markets.

It is well known that both the cultivar and the yeast strongly affect the wine quality [14]. Thus, in this work we assessed the impact of these two biotic factors on the pomegranate wine quality, by fermenting the juice of two pomegranate cultivars with two different commercial *Saccharomyces* yeasts strains.

This work can be considered as the first approach to the industrial production of pomegranate wine in Southern Italy: it poses the basis for both the future optimization of the technological process and the assessment of its economic potential, at local as well as international scale.

## 2. Materials and Methods

### 2.1. Collection of Pomegranate Fruits and Preparation of the Juice

Pomegranates were collected in commercial fields belonging to the company ICS s.r.l. The orchards are located in Salento, Apulia (Southern Italy; 40°19′44″ N–17°58′50″ E and 40°20′17″ N–17°59′31″ E). Two cultivars were considered for the experiment: “Jolly Red” (a seedless cultivar) and “Smith”. Fruits were collected at full ripening: 18 October 2020 and 25 October 2020 for “Smith” and “Jolly Red”, respectively. The next day after harvesting, juice was obtained by using a semiautomatic fruit squeezer (Video S1). The average fruit weight, the juice extraction yield, and the sugar content were measured in 18 replications of about 10 kg of fruit each.

After the addition of 80 mg L^−1^ potassium metabisulfite (Oeno METABISOLFITO DI POTASSIO, Enovys s.r.l., Peschiera del Garda, Italy), commercial white beet sugar was added until reaching ~21 °Brix, along with 10 mg L^−1^ Lallzyme HC pectolytic enzymes (Lallemand Inc., Montreal, QC, Canada) and 750 mg L^−1^ E-BENTHON EXTRA bentonite (Perdomini-IOC SPA, San Martino Buon Albergo, Italy), and the juice underwent a static decanting overnight at 11 °C.

### 2.2. Pomegranate Juice Fermentations

The next day, inocula of the commercial yeasts *Saccharomyces cerevisiae* Clos and *Saccharomyces cerevisiae* ex-*bayanus* EC1118 (both Lallemand Inc., Montreal, QC, Canada) were prepared and were added to the clarified juices at a concentration of 1.5 g L^−1^. The two yeasts were chosen due to their differences in oenological and physiological properties (Table S1). The inocula were prepared by rehydrating the yeasts in 50:50 warm water:pomegranate juice first, and then by progressively adding pomegranate juice to gradually adapt the yeasts to the fermentation conditions.

A total of 12 fermentations (2 cultivars × 2 yeasts, 3 replications each) of 21–22 L each were carried out in steel tanks. An amount of 0.5 g L^−1^ of yeasts nutrients (Oeno ACTIV NUTRIENTI, Enovys s.r.l., Peschiera del Garda, Italy) was added to each fermentation tank. During the fermentation process, juice density (using a hydrometer), yeast cell concentration (using a Burker cell-counter chamber), and juice temperature were measured daily. Superficial foam was removed when it appeared too abundant.

The fermentations were stopped when the density reached a value of ≤1, by adding potassium metabisulfite (100 mg L^−1^) to ensure the microbiological stability of the product. A racking allowed the lees separation. The obtained pomegranate wines were kept in filled 5 L bottles in the dark until further analyses.

The significance of the differences in juice density, temperature, and yeast concentrations between cultivars, yeasts, and their interaction were calculated by ANOVA repeated measures on fermentation days 1 to 8. Where appropriate, *t*-test was performed between groups at individual fermentation days.

### 2.3. Wine Analyses

Wines were analyzed 3 months after fermentation. A first screening of the pomegranate wine quality was obtained in collaboration with the laboratory of the Menhir Salento S.p.a. winery. pH was measured using a “pHmeter Sension + pH3” (HACH) with an electrode 5012 T. Free SO_2_ and total SO_2_ were obtained by iodometric titration. Iodine 0.01 N (Titolchimica), H_2_SO_4_ 95%–97% (Honeywell Fluka), NaOH (Honeywell Fluka), and starch indicator 1% (Titolchimica) were used. To quantify the free SO_2_, 2 mL of H_2_SO_4_ (diluted 1:4 in water) and 5 mL of starch indicator were added to 20 mL of sample. The volume (mL) of iodine 0.01 N necessary for the titration was multiplied by 16 to obtain the free SO_2_ (mg L^−1^). The total SO_2_ was quantified with the same procedure, but, in this case, the 20 mL of sample was previously reacted for 15 min with 2 mL of NaOH 4N.

Glucose + fructose quantification was obtained by enzymatic method. Samples were filtered using filter paper “Perfecte 2”—90 g m^—2^ (Cordenons SPA, Milano, Italy) and analyzed by a “Hyperlab Smart” analyzer (Steroglass, San Martino in Campo, Perugia, Italy).

#### 2.3.1. Organic Acids Analysis

Each sample of wine was filtered using a 0.2 μm filter, then 20 μL was injected into a Waters HPLC system composed of 600E pumps and a 996 Diode Array Detector (Waters Corporation, Milford, CT, USA). Separation was carried out using a Synergy Hydro RP column 80 Å, 4 µm, 250 mm × 4.6 mm (Phenomenex, Torrance, CA, USA). The mobile phases were 0.1% orthophosphoric acid in water (eluent A) and acetonitrile (eluent B). The gradient used was 0–18 min 100% A at 1 mL min^−1^ flow rate, then 18–18.3 min from 100% to 20% A, 18.3–19.5 min increasing flow rate to 1.4 mL min^−1^, then 19.5–22.5 min isocratic condition and 22.5–23 min from 20% to 100% A, and a final isocratic from 23 to 43 min. The chromatogram was acquired from 0 to 20 min, the last part of the gradient was used to clean and prepare the column for another injection. The detection of organic acids was done at λ = 214 nm. All the compounds were quantified using the external standard method with calibration curves for each organic acid, and the results were expressed as g·L^−1^.

#### 2.3.2. Total Phenolic Compounds Analysis

The determination of total phenol content (TPC) was performed by the Folin–Ciocalteu method according to Tarantino et al. [15]. Twenty microliters of wine was added to 980 µL of ddH_2_O and 100 µL of Folin–Ciocalteu reagent. After 3 min, 5% Na_2_CO_3_ solution was added, following incubation at room temperature for 60 min. The absorbance was read at 750 nm using a Cary 60 spectrophotometer (Agilent, Cernusco, Milano, Italy). The TPC was expressed as gallic acid equivalents (GAE) in mg·L^−1^ juice.

#### 2.3.3. Spectrophotometric Analyses

Juice samples were analyzed for color intensity and hue using a spectrophotometer. Each sample was placed into a 1 mm quartz cell, and a direct measurement of absorbance at 420, 520, and 620 nm was done. Color intensity was calculated as the sum of absorbance (Abs) at 420 + 520 + 620 nm, whereas hue was the ratio of the absorbance at 420 and 520 nm. Total anthocyanins were determined according to Gambacorta et al. [16] with slight modifications: samples were diluted with ethanol–hydrochloric acid solution and placed into a 10 mm cell, the absorbance spectrum was recorded in the range of 360–700 (VIS).

The A content was determined according to the following formula (1):

$$A=E_{maxvis}∙df∙16.7$$

where *E_maxvis_* = specific extinction coefficient at the maximum of visible region (~520 nm); *df* = dilution factor; 16.7 = value determined considering the molar extinction coefficient and molecular weight of cyanidin-3-O-glucoside (e = 26,900; MW = 449.2).

#### 2.3.4. Volatile Compounds Analysis

The volatile compounds were extracted by the solid-phase microextraction (SPME) technique, according to Trani et al. [17]. The samples were weighed (1 ± 0.05 g) into 20 mL vials containing 0.2 g/mL of NaCl (to increase the ionic strength), closed by a silicone/PTFE septum and an aluminum cap. All samples were added with internal standard (2-octanol) to perform a semi-quantitation. A mother solution obtained from the pure standard (Sigma Aldrich, Milan, Italy), with a concentration of 820 mg L^−1^, was diluted to reach a final concentration of 8.2 μg L^−1^, then 10 μL of this final dilution was added to the sample. Samples were loaded into an autosampler Triplus RSH (ThermoFisher Scientific, Rodano, Italy). Before extraction, stabilization of the headspace in the vial was obtained by equilibration for 10 min at 37 °C. The extraction was carried out using a divinylbenzene/carboxen/polydimethylsiloxane (DVB/CAR/PDMS) 50/30 mm SPME fiber assembly (Supelco, Bellefonte, PA, USA) at 37 °C for 15 min. The fiber was desorbed at 220 °C for 2 min in the injection port of the gas chromatograph, operating in split-less mode. The GC-MS analyses were performed using a Trace1300 gas chromatograph equipped with a mass spectrometer ISQ Series 3.2 SP1. The compounds were separated on a Thermo capillary column VF-WAX MS (60 m, 0.25 mm, 0.25 mm), under the following conditions: injection port temperature, 220 °C; oven temperatures, 40 °C for 0.1 min then 4 °C min^−1^ to 140 °C, 10 °C min^−1^ to 220 °C final isothermal for 7.5 min. Mass detector was set at the following conditions: detector voltage, 1700 V; source temperature, 250 °C; ionization energy, 70 eV; scan range, 33–200 amu. Tentative identification of the peaks was done by means of Xcalibur v2.0 software, in particular, Qual Browse, by matching their spectra with the reference mass spectra of NIST library. Semi-quantitation of the compounds was done by the internal standard method, and the amounts were expressed as micrograms of 2-octanol equivalents Liter^−1^.

### 2.4. Statistical Analysis

Two-way analysis of variance (ANOVA) with interactions was performed on the analytical data, considering the independent factors *cultivar* and *yeast* and their interaction *cultivar*×*yeast*.

Principal components analysis (PCA) and hierarchical cluster analysis (HCA) were performed. HCA was carried out after data standardization for each volatile compound; both wines and volatile compounds were clustered applying the Ward method and the squared Euclidean distance type.

Statistical analysis was performed using Origin 2021 (OriginLab, Northampton, MA, USA).

## 3. Results and Discussion

### 3.1. Collection of Pomegranate Fruits and Preparation of the Juice

The cultivar Jolly Red produced significantly heavier and juicier fruits than those of Smith. On the other hand, Smith sarcotesta had higher concentration in sugars (Table 1).

The sarcotesta juice is generally about 40% of the pomegranate fruit [18]. The extraction yield obtained with our semiautomatic extraction procedure was lower than this value; however, the extraction method significantly modifies the pomegranate juice yield [19]. In our conditions, a significant difference in the extraction yield was obtained, indicating better performances for the Jolly Red cultivar. Furthermore, this cultivar produces bigger fruits, and this could be an additional positive trait for the processing performances (e.g., time of harvesting and fruit manipulation in general).

The studied pomegranates were quite rich in total soluble solids (TSS): Gumienna et al. [18] reported an average of 15.6% of dry substances in the juice. The pomegranate (cultivar Wonderful) juice obtained by Mphahele et al. [19] had about 16.2 °Brix, similar to our Jolly Red. However, Smith had significantly higher TSS than that of Jolly Red.

### 3.2. Pomegranate Juice Fermentations

**Figure 1:**
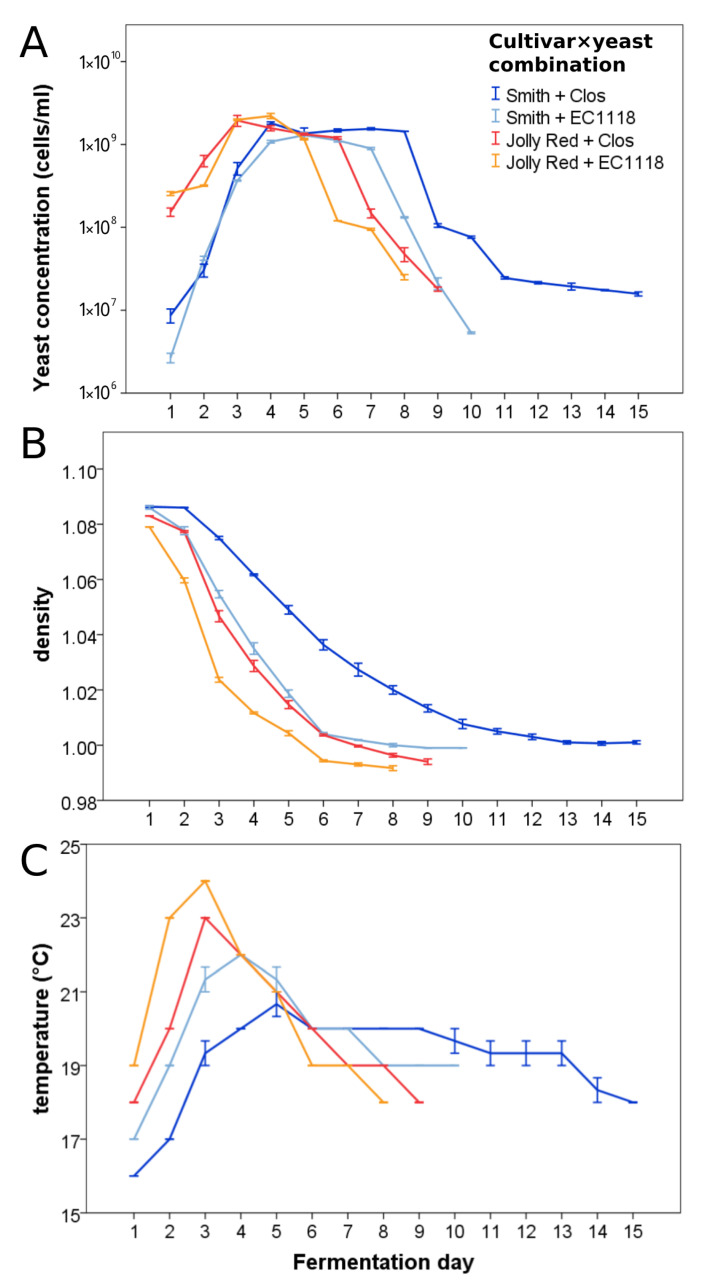
Dynamics of yeast concentrations (**A**), density (**B**), and temperature (**C**) during the fermentation process of pomegranate wines in the four cultivar×yeast combinations tested. Data are averages ± standard errors of three replicates per combination.

All combinations cultivar×yeast terminated the fermentation within 10 days upon reaching a density value of ≤1, except Clos on cultivar Smith, which slowed its fermentation process after day 9 and did not complete it totally (Figure 1). The latter was stopped on fermentation day 15 because the density curve was clearly steady (Figure 1B) and the temperature decreased (Figure 1C), indicating that the yeast cells became inactive or dead. In general, the fermentation of cultivar Jolly Red proceeded with a faster dynamic than that of Smith and yeast EC1118 was faster than Clos (Figure 1). Indeed, the faster combination was Jolly Red*EC1118, and the slower one, as mentioned above, was Smith*Clos (Figure 1).

Regarding fermentation monitoring (density and temperature), highly significant differences were found between both factors, *cultivar* and *yeast* (ANOVA repeated measures, *p* < 0.001), as well as their interaction (*p* < 0.001 for density and *p* = 0.003 for temperature) (Figure 1B,C). Regarding yeast concentration, highly significant differences were found between yeasts (ANOVA repeated measures, *p* < 0.001) and interaction cultivar×yeast (*p* = 0.002) (Figure 1A), but not between cultivars, when the data were analyzed by ANOVA repeated measures; however, the trends of the curves were clearly different, with Jolly Red being higher than Smith at days 1 (*t*-test, *p* < 0.001), 2 (*p* = 0.003), and 3 (*p* < 0.001), and Smith higher than Jolly Red at days 6 (*p* = 0.046), 7 (*p* = 0.001), and 8 (*p* = 0.050) (Figure 1A). No differences were found with *t*-test at days 4–5 (Figure 1A).

In our trial, we tested the effect of two factors on the process: the pomegranate cultivar and the yeasts. We found that both factors strongly influenced the fermentation process and that there was also a significant interaction between them. This was in contrast with the similar patterns found by Berenguer et al. [20], who fermented the combination of two pomegranate cultivars with three yeasts. Maybe this difference is due to the fact that we used two very different pomegranate cultivars and two very different commercial yeasts, while the three yeasts used by Berenguer and colleagues were all similar. 

The slower fermentation dynamics observed in Smith could be due to the more inhospitable medium according to Table 2 (e.g., higher acid concentration with consequent lower pH, higher phenolic contents, higher proportion of free SO_2_ with respect to total SO_2_, and higher final concentration in acetic acid). Despite this, the yeasts better completed the fermentation in these conditions, as demonstrated by the significantly lower residual sugars in the Smith wines. Considering the two yeasts, the greater difficulties of Clos compared to EC1118 are highlighted first of all by the higher concentration of residual sugars at the end of the fermentation. Nonetheless, also the higher acidification, SO_2_ complexation, and acetic acid production confirmed the difficulties of Clos. 

The dynamic of the fermentation process observed by Berenguer and colleagues appears similar to the ones found in our work, with most of the parameters reaching their final values within 10 days. In another work on optimization of parameters for pomegranate wine fermentation in India [21], the authors consistently found residual sugars; in contrast, for three out of the four cultivar×yeast combinations we tested, no notable residual sugar were found (see Table 2 below), thus demonstrating that our conditions were more suitable for completion of the pomegranate wine fermentation process.

### 3.3. Wine Characterization

#### 3.3.1. Basic Parameters

The cultivar, the yeast, and the cultivar×yeast interaction significantly affected the wine pH (Table 2). In particular, lower pH was obtained with the cultivar Smith, and with the yeast Clos. Despite the same amount of potassium metabisulfite being added to all of the fermentation tanks, the wines obtained by the yeast Clos had a lower total SO_2_ content. The same trend, but with a more pronounced effect, was observed concerning the free SO_2_. Considering this parameter, the cultivar also produced significant differences, with lower concentrations in Jolly Red wines. The residual sugars were significantly affected by the yeast and by the cultivar, with higher values in the wines fermented by the yeast Clos, and in the wines obtained by the cultivar Jolly Red.

The cultivar, the yeast, and the cultivar×yeast interaction significantly affected the wine pH (Table 2). In particular, lower pH were obtained with the cultivar Smith, and with the yeast Clos. Despite the same amount of potassium metabisulfite being added in all the fermentation tanks, the wines obtained by the yeast Clos had a lower total SO_2_ content. The same trend, but with a more pronounced effect, was observed concerning the free SO_2_. Considering this parameter, the cultivar also produced significant differences, with lower concentrations in Jolly Red wines. The residual sugars were significantly affected by the yeast and by the cultivar, with higher values in the wines fermented by the yeast Clos, and in the wines obtained by the cultivar Jolly Red. 

#### 3.3.2. Organic Acids

**Figure 2:**
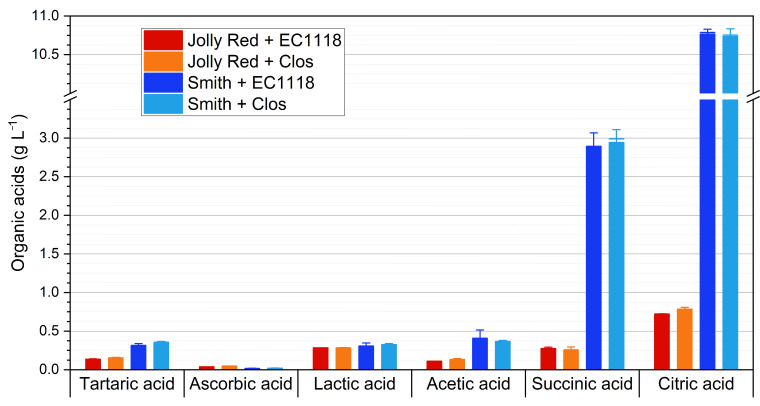
Contents of organic acids of the pomegranate wines from the four cultivar×yeast combinations tested. Data are averages ± standard deviations of three replicates per combination.

Figure 2 reports the amounts of organic acids detected in the pomegranate wines, while Table 3 reports the results of ANOVA. Citric acid is reported as the most abundant organic acid in pomegranate juices and wines [20,22,23,24,25]. The organic acid profile of wines showed the results of the lactic acid bacteria. In fact, malic acid was not detected, despite being the second organic acid in pomegranate juices as regards abundance [25,26], while lactic acid was found, indicating the occurrence of malolactic fermentation. Malolactic fermentation, in fact, consists of the decarboxylation of malic acid, obtaining lactic acid as final product. Indeed, the two yeasts used are considered as neutral (EC1118) or even positive (Clos) for the development of malolactic bacteria (Table S1).

The organic acid profile of the wines also included ascorbic acid and another compound that was identified as tartaric acid, based on the retention time of the standard compound. The literature reports contradictory findings regarding the presence of tartaric acid in pomegranate juices and wines. Lan et al. [27] described tartaric acid formation and decrease during pomegranate winemaking, with a maximum amount of 1.83 g L^−1^. Mena et al. [22] reported tartaric acid amounts in pomegranate varietal wines in the range ~0.2–0.6 g L^−1^, undergoing minimal variations during winemaking. As regards pomegranate juices, tartaric acid contents reported in literature range from trace levels to 2.83 g L^−1^ [26,28,29,30]. In this regard, an issue was raised by Ehling and Cole [23], who reported the risk of routine analytical methods to give false positives/negatives. They applied a method based on stable isotope dilution liquid chromatography–tandem mass spectrometry to twelve pomegranate juices and reported tartaric acid at concentrations in the range 1–5 mg L^−1^. Higher amounts (67–380 mg L^−1^) were found in four commercial juices but were attributed by the authors to grape juice adulteration. Lantzouraki et al. [31] investigated ten pomegranate semi-dry red wine samples from Wonderful variety, aging for 1 year in oak barrels, and did not detect tartaric acid when applying HPLC-PDA-ESI-MS^n^ analysis. 

In light of such uncertainty, it seems that further investigation is required considering the identification, detection, and concentrations, as well as variability of tartaric acid in pomegranate juices and wines.

Significant differences were observed among the organic acid profiles of the pomegranate wines. *Cultivar* was the most influencing variable. The most relevant difference regarded the content of citric acid was that wines from Jolly Red cv. contained less than 0.8 g L^−1^ of citric acid, while wines from Smith cv. contained above 10 g L^−1^. Considering that citric acid is characterized by high perceived sourness [32], attention should be paid to the observed difference for possible implications on the sensory characteristics of the wines.

The contents of succinic acid reported in the acidic profile of pomegranate juices ranges from 0.00 to 1.54 g L^−1^ [26,28,29]. Higher values were found in Smith wines. This could be attributed to cultivar variability, as well as to fermentation. In fact, variations of succinic acid were reported as a consequence of pomegranate winemaking [33]. The activity of lactic acid bacteria could also have contributed to reach such high amounts of succinic acid, considering that it has been reported as a product of citrate metabolism in lactic acid bacteria [34].

The levels of acetic acid were below critical thresholds (the maximum admitted level, according to OIV, is 1.2 g L^−1^) [35]. Acetic acid levels in Smith wines were three-fold higher than those in Jolly Red wines, while no significant effect of the yeast was observed. This seems to confirm the role of citrate metabolism of lactic acid bacteria in the genesis of this organic acid. Further investigation is required to explore the potential of malolactic fermentation in modulating the chemical and sensory profile of pomegranate wines.

#### 3.3.3. Phenolic Compounds and Spectrophotometric Analysis

**Figure 3:**
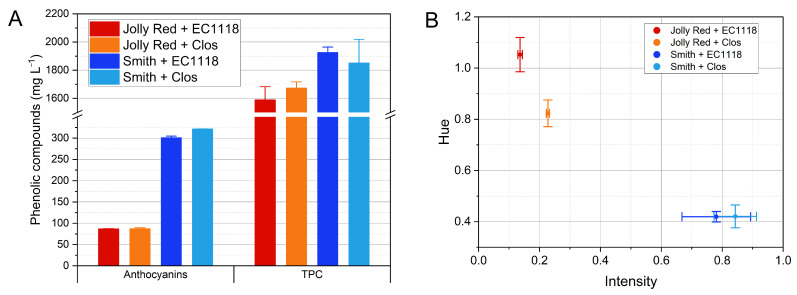
Contents of total anthocyanins and total phenolic compounds (TPC) (**A**) and color parameters (**B**) of the pomegranate wines from the four cultivar×yeast combinations tested. Data are averages ± standard deviations of three replicates per combination.

The pomegranate wines showed contents in TPC ranging from 1588 to 1924 mg GAE L^−1^ (Figure 3A and Table 4). The levels observed fell in the range reported in literature for pomegranate wines (704–3830 mg GAE L^−1^) [22,31,36]. Wines from cv. Smith presented higher contents of TPC compared with wines from Jolly Red. No significant effect could be attributed to the yeast.

The cultivars were significantly different for total anthocyanin contents. Wines from Jolly Red were less pigmented (87 mg L^−1^ with both yeasts). Wines from Smith presented much higher levels of anthocyanins, slightly above 300 mg L^−1^, with higher levels with Clos yeasts. Kokkinomagoulos et al. [36] found contents of anthocyanins in pomegranate wines fermented with different yeast strains in the range 69.6–78.7 mg L^−1^, while Lan et al. [27] reported an anthocyanin content of 23.02 mg L^−1^ after winemaking of a pomegranate juice. According to Mena et al., anthocyanins ranged from 14 to 62 mg L^−1^ in three pomegranate varietal wines [22]. Rios-Corripio et al., instead, reported much lower anthocyanin contents in a fermented pomegranate juice (around 2 mg L^−1^) [37]. Finally, Berenguer et al. [20] reported an effect of the yeast strain on the levels of anthocyanins in pomegranate wines, which were in the range 91.26–141.76 mg L^−1^. Our results agree with those reported in the literature and indicate a variability of anthocyanin contents in pomegranate wines that could be exploited by developing varietal wines differing for the degree of pigmentation. Moreover, another source of variability in anthocyanin contents can be found in juice preparation, which could significantly differ for extraction procedure, juice clarification, and possible pasteurization.

Data on color of the wines are reported in Figure 3B and Table 4. Color intensity fell in the typical range of rosé wines, with wines from cv. Smith having higher color intensity [38,39,40]. Color hue, determined as the ratio between absorbances at 420 and 520 nm, was lower in Smith wines, which showed a bright pink-reddish hue, while higher values were observed in Jolly Red wines, having pale pink hue. Such differences could be due to differences in both anthocyanin contents and pH values. Yeasts had a significant effect on the hue of wines: fermentation by Clos led to lower hue values, especially in Jolly Red. This could be related to the lowering effect of Clos yeasts on wine pH, with the consequent increase of anthocyanin color intensity.

#### 3.3.4. Volatile Compounds

A total of 46 volatile compounds were identified in the wines (Figure S1 and Table 5). Alcohols and esters were the most abundant classes of compounds, deriving from fermentation. Isoamyl alcohol and 2-phenylethanol were the alcohols found in highest amounts, while the most abundant esters were isoamyl acetate and phenylethyl-acetate (among acetates), ethyl hexanoate, and octanoate and decanoate (among ethyl esters). Similar volatile profiles were reported in the literature [27,36,41,42]. Several significant differences were however observed in the levels of volatile compounds, due to both *Cultivar* and *Yeast* variables. Wines from Jolly Red were richer in esters. Further research is needed to assess whether these differences could underlie different sensory properties related to fruity perception. The effects of yeast strains also involved differences in the amounts of several esters, suggesting quite different flavor profiles.

**Figure 4:**
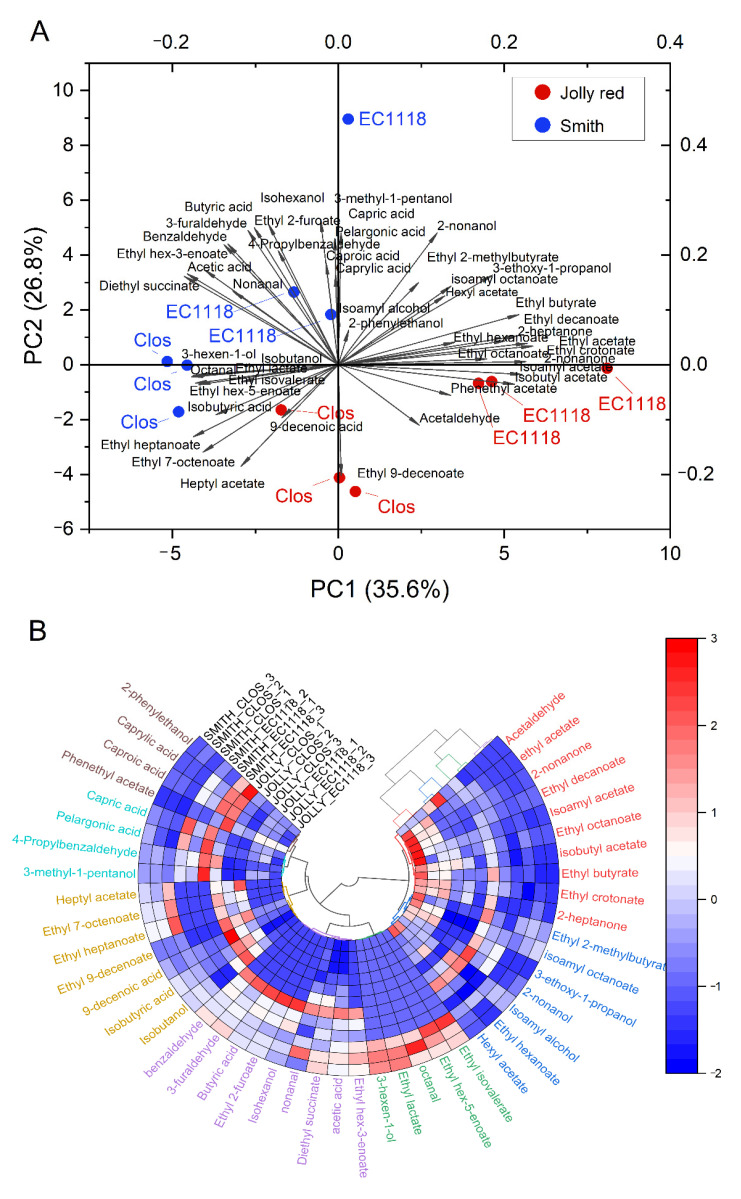
Results of the multivariate analysis of the volatile profiles of the pomegranate wines from the four cultivar×yeast combinations tested: biplot of the first two principal components of PCA (**A**) and polar heatmap with a circular dendrogram deriving from a hierarchical cluster analysis (**B**).

A comprehensive evaluation of the volatile patterns was obtained by applying multivariate analysis. Figure 4A reports the biplot of the principal components analysis (PCA).

The first two principal components deriving from the PCA accounted for 52.4% of total variability. The twelve pomegranate wines were grouped on the plane of PC1 and PC2, according to both cv. and yeast. Fermentation by EC1118 seemed to emphasize the varietal differences: the wines fermented by Clos, in fact, clustered closer on the plane of the PCA. 

In Figure 4B, a polar heatmap with a circular dendrogram deriving from a hierarchical cluster analysis is reported and allows to characterize the volatile patterns. In fact, wines were clusterized in four homogeneous groups, indicating that both cv. and yeast conferred specific features to the volatile profile. Moreover, volatile compounds were clusterized in seven groups characterized by a certain homogeneity related to the chemical nature and origin of volatiles. The first two clusters (analyzing clockwise the circular heatmap) included acetate esters (also known as *fruit esters* [43]), fatty acid ethyl esters, and the two ketones found in the wines. This cluster characterized the wines fermented with EC1118, particularly from Jolly Red. Esters are typical products of fermentation related to secondary aroma of wines [44,45] and are mainly responsible for fruity notes. The compounds in this cluster have been related to perceptions such as apple (isobutyl-acetate, ethyl-2-methylbutyrate, ethyl-hexanoate, hexyl-acetate, isoamyl-hexanoate), banana (isoamyl-acetate, isobutyl-acetate, isoamyl-butyrate, ethyl-hexanoate), pineapple (ethyl-acetate, ethyl-butyrate, isoamyl-butyrate, isoamyl-hexanoate), and pear (isobutyl-acetate, isoamyl-acetate, ethyl-decanoate) [42,45]. The possible effect on the sensory properties of the wines needs to be assessed by further research. The third and fourth clusters of volatiles characterized the wines obtained from cv. Smith, and particularly those fermented with Clos yeasts. The volatile compounds belonging to these clusters included unsaturated six-carbon alcohols and esters together with two aldehydes (i.e., octanal and nonanal). Both C6 unsaturated compounds and C8/C9 aldehydes derive from enzymatic and chemical fatty acid oxidation [43,46]. This could be the reason why they characterized wines from cv. Smith, in which juice extraction could have also caused the release of lipids from the seeds. On the contrary, this did not happen in wines from Jolly Red, which is a seedless cultivar. The effect of unsaturated fatty acids deriving from pomegranate seeds is confirmed by studies investigating the effect of unsaturated fatty acid supplementation during winemaking. Recently, Liu et al. [47] reported that unsaturated fatty acid supplementation determined an increase of C6 alcohols and a decrease of medium-chain fatty acids and acetate esters. Moreover, they observed some strain-related effects, as observed in our study. Ethyl lactate confirms the occurrence of malolactic fermentation [45], and the concurrent presence of diethyl succinate could strengthen the hypothesis of succinate formation by lactic acid metabolism. The fifth cluster included unsaturated medium-chain fatty acids (7-octenoate and 9-decenoate) and a corresponding ester. Such compounds are commonly found in wines and other fermented beverages, including pomegranate wine [27,48]. Though their formation is not clearly defined, Clos yeasts seemed to express at higher levels such metabolic pathways. The last two clusters grouped several relevant compounds, including 2-phenylethanol and its acetate ester, potentially related to floral and honey perception [44], as well as medium-chain fatty acids (C6–C8–C10). No clear relation could be inferred with either cultivar or yeast.

Sensory analysis is required to confirm whether chemical clustering of wines corresponds to sensory differentiation. In this case, the significant effect of variables such as cultivar, yeast strain, and seed involvement in winemaking, on the volatile profile of pomegranate wines would indicate the possibility to modulate their sensory properties according to preferences or specific winemaking styles and objectives.

## 4. Conclusions

In general, it was possible to achieve a complete fermentation of pomegranate juice with different experimental settings. This work poses the bases for future perfectioning of the fermentation process, thus opening new opportunities regarding under exploited market opportunities. 

The combination of different cultivars and yeasts could be a powerful leverage to tailor pomegranate wines with desired chemical profiles and, consequently, sensory properties, including the organic acid profile, as well as color and volatile pattern. Moreover, some issues related to the influence of the chemical environment on yeast metabolism were highlighted and require further research in order to provide knowledge tools for an optimal management of pomegranate winemaking.

Considering the current interest in pomegranate beverages due to their well-known beneficial properties, pomegranate wine is a promising candidate as a new commercial standard product for large consumption in Western Countries. Further studies will be necessary to investigate the perceptions of new potential consumers for these products in Western Countries, from both the organoleptic and commercial points of view.

## Figures and Tables

**Table 1:** Fruit characterization—comparison between the two cultivars. Results of ANOVA are also reported (*p*-values *)

	Average Fruit Weight (kg)		Extraction Yield (%)		Total Soluble Solids (°Brix)	
	Mean	SD	Mean	SD	Mean	SD
Jolly Red	0.275	0.004	31.733	0.236	16.167	0.09
Smith	0.211	0.004	29.624	0.324	17.028	0.086
ANOVA						
Significance	**<0.001**		**<0.001**		**<0.001**	

* Significant values (*p* < 0.05) are in bold (*n* = 3).

**Table 2:** Wine characterization (average values ± standard errors) at the end of the pomegranate winemaking process (3 months after end of fermentation). The significance of the cultivar, yeast, and cultivar×yeast interaction effects are also reported (*p*-values *).

	pH		Free SO_2_ (mg L^−1^)		Total SO_2_ (mg L^−1^)		Glucose + Fructose (g L^−1^)	
	Mean	SE	Mean	SE	Mean	SE	Mean	SE
Smith + Clos	3.12	0.01	45.67	0.33	55.00	0.58	2.41	0.60
Smith + EC1118	3.14	0.00	54.67	0.67	64.67	2.91	0.29	0.04
Jolly Red + Clos	3.51	0.00	21.00	1.53	49.33	0.67	6.84	0.54
Jolly Red + EC1118	3.64	0.01	27.00	1.53	58.67	1.86	1.60	0.38
	ANOVA							
Cultivar Significance	**<0.001**		**<0.001**		**0.011**		**<0.001**	
Yeast Significance	**<0.001**		**<0.001**		**0.001**		**<0.001**	
Cultivar×Yeast Significance	**<0.001**		0.226		0.928		**0.008**	

* Significant values (*p* < 0.05) are in bold (*n* = 3).

**Table 3:** Results of two-way analysis of variance on organic acids contents of pomegranate wines (*p*-values *).

	Cultivar	Yeast	Cultivar×Yeast
Tartaric acid	**<0.001**	0.414	**0.047**
Ascorbic acid	**<0.001**	0.105	**<0.001**
Lactic acid	**0.033**	0.493	0.519
Acetic acid	**<0.001**	0.355	0.769
Succinic acid	**<0.001**	0.653	0.911
Citric acid	**<0.001**	0.290	0.768

* Significant values (*p* < 0.05) are in bold (*n* = 3).

**Table 4:** Results of two-way analysis of variance on phenolic compounds and color of pomegranate wines (*p*-values *).

	Cultivar	Yeast	Cultivar×Yeast
*Phenolic compounds*			
TPC **	**0.002**	0.928	0.212
Total anthocyanins	**<0.001**	**<0.001**	**<0.001**
*Color*			
Intensity	**<0.001**	0.080	0.716
Hue	**<0.001**	**0.004**	**0.004**

* Significant values (*p* < 0.05) are in bold (n = 3); ** Total phenolic compounds.

**Table 5:** Results of the SPME-GC/MS analysis of the headspace of the pomegranate wines. Amounts are expressed in micrograms of 2-octanol equivalents Liter^−1^. The results of ANOVA are also reported.

	RT *	Jolly Red + EC1118		Jolly Red + Clos		Smith + EC1118		Smith + Clos		ANOVA Results (*p*-Values) **		
		Mean	*SD*	Mean	*SD*	Mean	*SD*	Mean	*SD*	Cultivar	Yeast	Cultivar×Yeast
*Aldehydes*												
Acetaldehyde	4.83	0.81	*0.04*	1.23	*0.60*	0.52	*0.30*	0.36	*0.01*	**0.017**	0.523	0.180
Octanal	20.8	0.00	*0.00*	0.00	*0.00*	0.00	*0.00*	0.39	*0.16*	**0.003**	**0.003**	**0.003**
Nonanal	25.36	0.97	*0.15*	1.35	*0.73*	1.78	*1.08*	2.14	*1.07*	0.139	0.475	0.985
3-furaldehyde	28.56	0.00	*0.00*	0.00	*0.00*	0.34	*0.19*	0.30	*0.06*	**<0.001**	0.733	0.733
Benzaldehyde	31.07	0.00	*0.00*	0.00	*0.00*	0.80	*0.25*	0.63	*0.10*	**<0.001**	0.298	0.298
4-Propylbenzaldehyde	42.73	1.42	*0.01*	2.08	*1.77*	3.42	*1.07*	2.07	*0.27*	0.136	0.582	0.135
Total		3.21	*0.16*	4.66	*1.96*	6.87	*2.85*	5.90	*1.28*	0.051	0.827	0.290
*Alcohols*												
Isobutanol	12.41	18.57	*0.78*	32.34	*7.49*	21.31	*8.37*	27.30	*0.96*	0.735	**0.016**	0.267
Isoamyl alcohol	17.11	765.38	*23.25*	783.31	*438.01*	636.96	*595.21*	768.34	*45.17*	0.746	0.736	0.798
Isohexanol	21.77	0.00	*0.00*	0.00	*0.00*	0.92	*0.39*	0.40	*0.11*	**<0.001**	0.056	0.056
3-methyl-1-pentanol	22.34	1.13	*0.27*	0.90	*0.57*	1.96	*0.79*	1.13	*0.10*	0.107	0.108	0.335
3-ethoxy-1-propanol	24.51	1.74	*0.05*	0.00	*0.00*	1.34	*0.40*	0.00	*0.00*	0.124	**<0.001**	0.124
3-hexen-1-ol	24.88	0.00	*0.00*	0.00	*0.00*	0.00	*0.00*	1.94	*0.24*	**<0.001**	**<0.001**	**<0.001**
2-nonanol	30.48	0.82	*0.20*	0.41	*0.10*	1.00	*0.41*	0.40	*0.09*	0.541	**0.007**	0.530
2-phenylethanol	45.71	377.38	*9.23*	514.26	*254.25*	422.26	*142.19*	306.38	*34.53*	0.364	0.904	0.174
Total		1165.02	*32.46*	1331.20	*670.92*	1085.76	*744.64*	1105.89	*80.84*	0.614	0.757	0.808
*Acetate esters*												
Ethyl acetate	6.81	76.42	*10.10*	50.19	*3.69*	54.56	*6.51*	44.69	*0.62*	**0.005**	**0.001**	0.054
Isobutyl acetate	9.84	0.90	*0.16*	0.56	*0.32*	0.36	*0.30*	0.23	*0.04*	**0.012**	0.123	0.469
Isoamyl acetate	13.72	106.46	*51.76*	56.62	*9.48*	39.46	*22.05*	22.46	*6.23*	**0.016**	0.078	0.351
Hexyl acetate	20.02	0.91	*0.46*	0.60	*0.20*	0.78	*0.50*	0.59	*0.11*	0.746	0.259	0.792
Heptyl acetate	24.45	0.00	*0.00*	0.40	*0.18*	0.00	*0.00*	0.29	*0.14*	0.394	**<0.001**	0.394
Phenethyl acetate	42.25	104.21	*6.38*	105.90	*57.97*	47.39	*14.79*	21.77	*2.69*	**0.004**	0.511	0.455
Total		288.89	*55.47*	214.28	*54.55*	142.54	*41.05*	90.03	*3.59*	**<0.001**	**0.037**	0.675
*Ethyl esters*												
Ethyl butyrate	10.59	7.87	*1.68*	4.71	*0.23*	5.57	*2.36*	3.29	*0.42*	0.059	**0.013**	0.615
Ethyl 2-methylbutyrate	11.11	1.46	*0.94*	0.46	*0.10*	1.12	*0.95*	1.11	*0.09*	0.695	0.227	0.239
Ethyl isovalerate	11.67	0.00	*0.00*	0.00	*0.00*	0.00	*0.00*	0.15	*0.06*	**0.001**	**0.001**	**0.001**
Ethyl crotonate	15.41	0.41	*0.02*	0.18	*0.07*	0.20	*0.08*	0.06	*0.05*	**0.001**	**<0.001**	0.253
Ethyl hexanoate	18.34	52.97	*20.17*	44.96	*18.26*	38.58	*28.63*	29.55	*12.74*	0.249	0.497	0.967
Ethyl hex-5-enoate	20.32	0.00	*0.00*	0.00	*0.00*	0.00	*0.00*	0.22	*0.07*	**<0.001**	**<0.001**	**<0.001**
Ethyl hex-3-enoate	21.32	0.00	*0.00*	0.00	*0.00*	0.22	*0.12*	0.28	*0.05*	**<0.001**	0.485	0.485
Ethyl heptanoate	22.69	0.00	*0.00*	0.18	*0.07*	0.00	*0.00*	0.27	*0.11*	0.289	**<0.001**	0.289
Ethyl lactate	23.14	0.00	*0.00*	0.00	*0.00*	0.00	*0.00*	0.61	*0.11*	**<0.001**	**<0.001**	**<0.001**
Ethyl octanoate	27.05	225.17	*154.14*	128.64	*55.10*	109.05	*76.25*	89.35	*21.07*	0.177	0.300	0.485
Ethyl 7-octenoate	29.22	0.00	*0.00*	0.62	*0.15*	0.00	*0.00*	0.63	*0.23*	0.938	**<0.001**	0.938
Ethyl 2-furoate	34.94	0.00	*0.00*	0.00	*0.00*	1.71	*0.90*	1.00	*0.27*	**0.001**	0.223	0.223
Ethyl decanoate	35.36	246.63	*104.96*	40.69	*10.03*	96.15	*41.57*	47.57	*5.56*	0.060	**0.005**	**0.043**
Ethyl 9-decenoate	37.41	66.08	*7.32*	194.20	*30.99*	11.15	*1.49*	56.60	*5.31*	**<0.001**	**<0.001**	**0.002**
Total		600.59	*284.87*	414.64	*93.96*	263.77	*150.91*	230.69	*33.70*	**0.028**	0.294	0.455
*Other esters*												
Isoamyl octanoate	36.14	3.16	*0.77*	0.00	*0.00*	1.99	*0.53*	1.68	*0.41*	0.411	**<0.001**	**0.001**
Diethyl succinate	36.88	0.00	*0.00*	2.24	*1.74*	4.11	*1.70*	4.14	*1.12*	**0.005**	0.180	0.191
Total		3.16	*0.77*	2.24	*1.74*	6.10	*2.14*	5.82	*1.51*			
*Ketones*												
2-heptanone	16.16	0.30	*0.04*	0.10	*0.10*	0.09	*0.16*	0.03	*0.05*	**0.037**	**0.043**	0.247
2-nonanone	25.12	1.26	*0.47*	0.17	*0.07*	0.34	*0.10*	0.08	*0.07*	**0.007**	**0.001**	**0.018**
Total		1.57	*0.50*	0.26	*0.15*	0.43	*0.13*	0.11	*0.02*	**0.003**	**<0.001**	**0.013**
*Acids*												
Acetic acid	28.09	3.23	*0.12*	6.20	*1.06*	9.52	*3.01*	9.50	*0.66*	**<0.001**	0.156	0.151
Isobutyric acid	32.77	0.00	*0.00*	1.42	*0.34*	0.50	*0.16*	0.84	*0.12*	0.723	**<0.001**	**0.002**
Butyric acid	35.17	0.00	*0.00*	0.00	*0.00*	0.81	*0.36*	0.48	*0.03*	**<0.001**	0.160	0.160
Caproic acid	43.24	21.37	*5.00*	27.35	*17.81*	37.54	*11.10*	18.08	*2.07*	0.597	0.313	0.076
Caprylic acid	50.46	257.37	*64.35*	336.07	*232.71*	418.63	*146.95*	194.73	*23.26*	0.906	0.401	0.102
Pelargonic acid	53.82	1.78	*0.41*	1.71	*0.34*	2.69	*0.85*	1.59	*0.36*	0.235	0.094	0.129
Capric acid	57.04	45.06	*11.26*	23.16	*15.52*	116.10	*44.50*	31.60	*9.37*	**0.024**	**0.006**	0.059
9-decenoic acid	58.97	2.33	*0.91*	31.75	*25.12*	4.41	*2.35*	9.16	*3.19*	0.200	**0.049**	0.132
Total		331.13	*81.05*	427.67	*292.51*	590.21	*195.21*	265.98	*34.64*	0.654	0.308	0.079

* Retention time (min); ** Significant values (*p* < 0.05) are in bold (n = 3).

## Data Availability

The data presented in this study are available on request from the corresponding author.

## Supplementary Materials

The following are available online at https://www.mdpi.com/article/10.3390/foods10081913/s1, Figure S1: Sample chromatograms of SPME-GC/MS analysis of the pomegranate wines, Table S1: Oenological and physiological properties of the yeasts used in this work, as reported by the manufacturer, Video S1: The squeezer used in this work as operated to obtain the pomegranate juice.
