# Supplementary material for: Dynamics of the Fermentation Process and Chemical Profiling of Pomegranate (Punica granatum L.) Wines Obtained by Different Cultivar×Yeast Combinations
Source: Foods. 2021 Aug 18;10(8):1913. doi: 10.3390/foods10081913 (PMC8392717; doi:10.3390/foods10081913)
Supplement: Supplementary file 1 [file foods-10-01913-s001.zip › Figure S1.pdf]

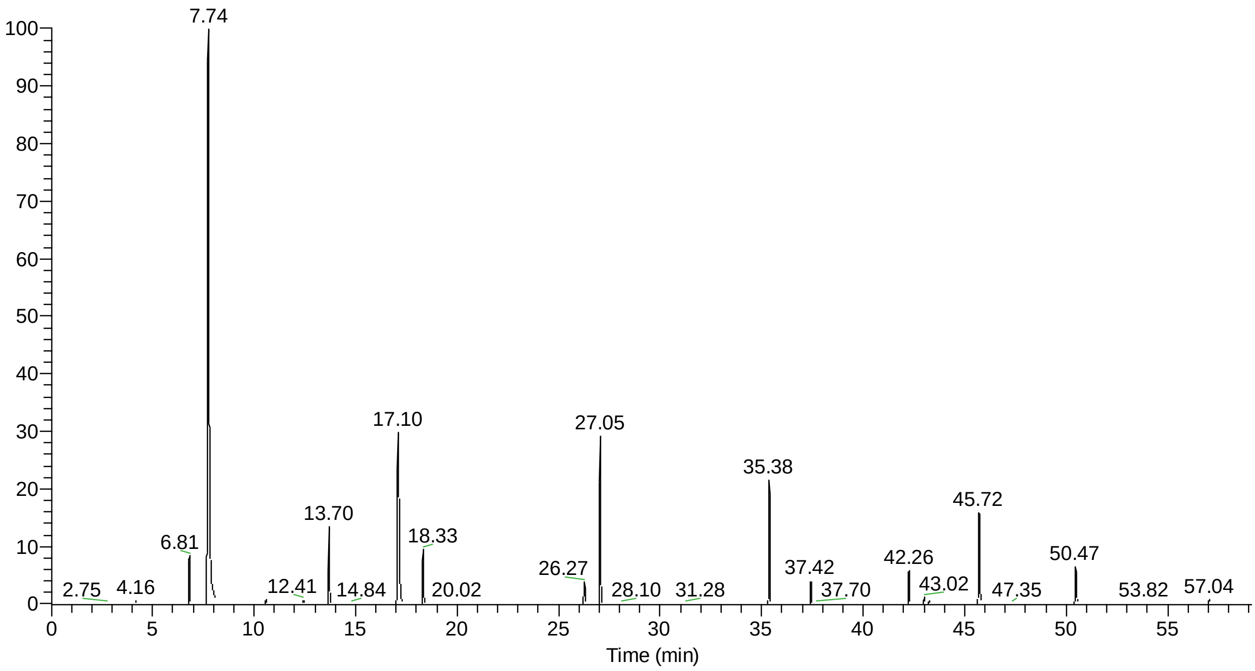

JOLLY RED – EC1118

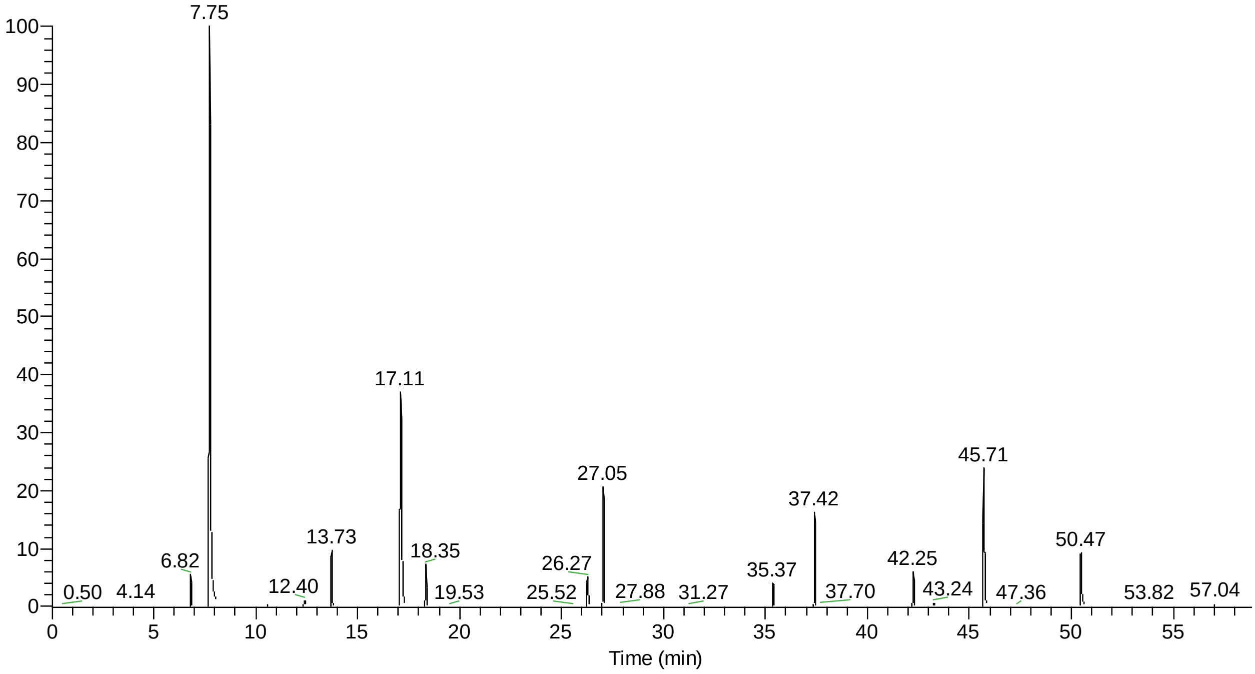

JOLLY RED – CLOS

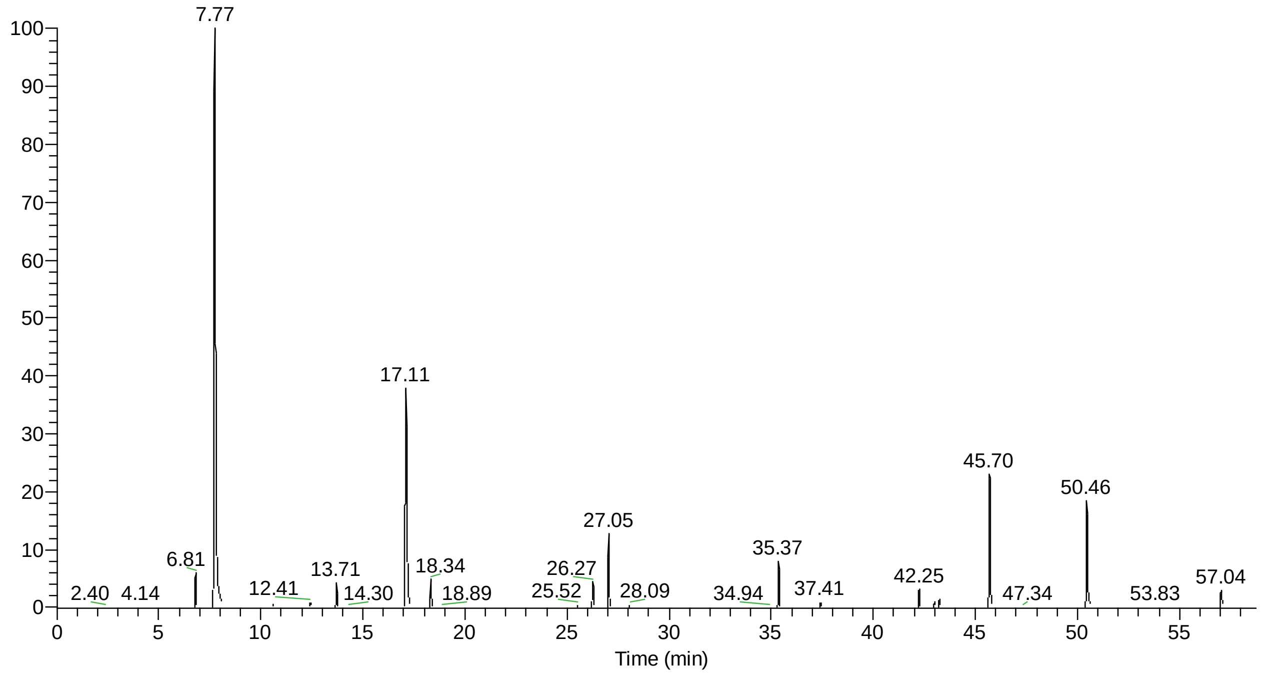

SMITH – EC1118

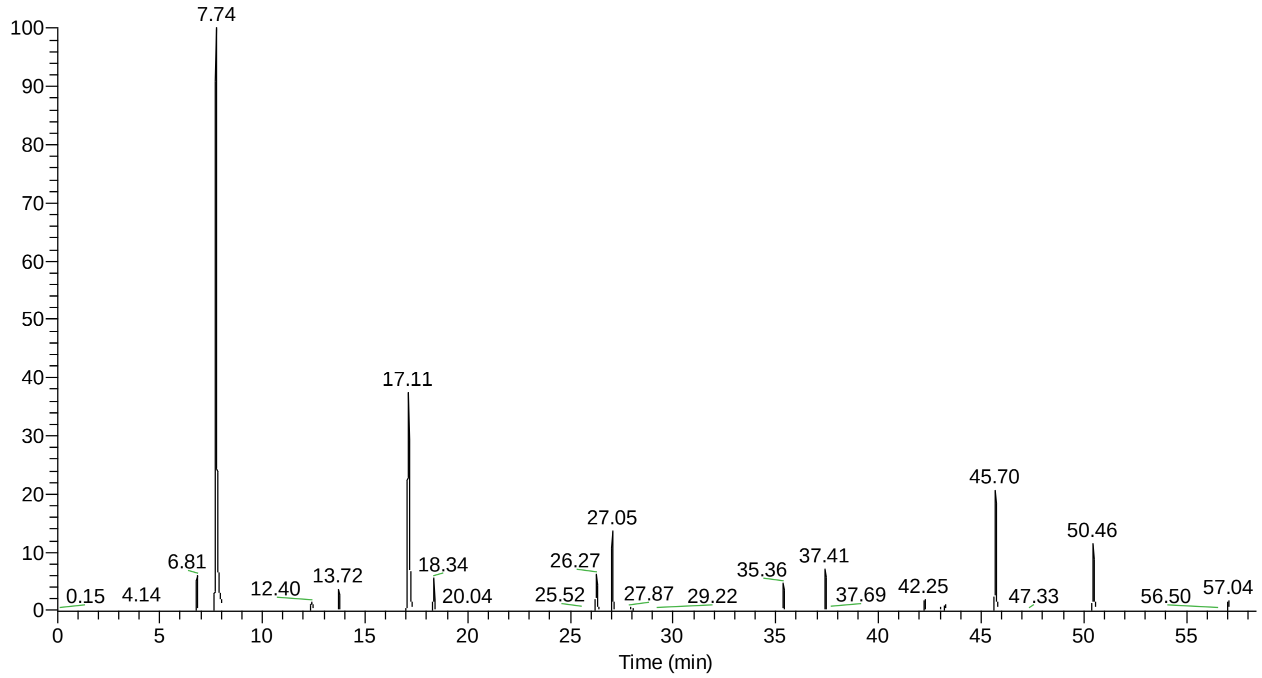

SMITH – CLOS

Figure S1. Sample chromatograms of SPME-GC/MS analysis of the pomegranate wines.
