# Supplementary material for: Dynamics of the Fermentation Process and Chemical Profiling of Pomegranate (Punica granatum L.) Wines Obtained by Different Cultivar×Yeast Combinations
Source: Foods. 2021 Aug 18;10(8):1913. doi: 10.3390/foods10081913 (PMC8392717; doi:10.3390/foods10081913)
Supplement: Supplementary file 1 [file foods-10-01913-s001.zip › Table S1_Characteristics of yests Clos and EC1118.pdf]

foods

MDPI

Tab. S1. Oenological and physiological properties of the yeasts used in this work, as reported by the manufacturer (Lallemand Inc., Montreal, Canada; www.lallemandwine.com/en/uk-scandinavia/products/catalogue/wine-yeasts/20/lalvin-ec1118--prise-de-mousse/ and www.lallemandwine.com/fr/france/produits/catalogue/levures-oenologiques/12/lalvin-clos/ - accessed on 2021/08/07). nd.: not determined

Property	*Saccharomyces cerevisiae* Clos	*Saccharomyces cerevisiae* (ex-*bayanus*) EC1118
Fermentation temperature range (°C)	10-30	13-35
Sugar preference	Glucosophilic	Fructosophilic
Killer toxin	Yes	Yes
Resistance to Ethanol	17%	≤18%
Latency (lag phase)	Short/Very short	Short/Very short
Fermentation vigor	High	High
Fermentation dynamic	Fast and regular	Fast
N requirements	Low/Medium	Low
O_2 requirement	nd.	Low
Acetic acid production	Low	Low
Acetaldehyde production	Moderate	Low
H_2S production	Low	Very low
SO_2 production	Low	Moderate/High
Glycerol production	High	High
Foam production	nd.	Low
Resistance to SO_2	High	nd.
Interaction with malolactic bacteria	Positive	Neutral
Impact on organoleptic properties	High	Low
Suitable for secondary fermentation	No	Yes
Flocculation	No	Yes
Prevalence over native yeasts	nd.	High
